# BENDI: Improving Cognitive Assessments in Toddlers and Children with Down Syndrome Using Stealth Assessment

**DOI:** 10.3390/children10121923

**Published:** 2023-12-13

**Authors:** Marcela Tenorio, Paulina S. Arango, Andrés Aparicio

**Affiliations:** 1 Universidad de los Andes, Santiago 7620001, Chile; parango@uandes.cl; 2 Millennium Institute for Care Research (MICARE), Santiago 8370146, Chile; aaparicio@micare.cl

**Keywords:** tablets, games, down syndrome, cognition, assessment

## Abstract

Cognitive assessment is a fundamental step in diagnosing intellectual and developmental disabilities, designing interventions, and evaluating their impact. However, developed and developing countries have different access to tools designed for these purposes. Our goal was to develop a battery for cognitive assessment mediated by digital technology that allows the exploration of cognitive domains (inhibitory control, attention, motor ability, and context memory) in children with Down Syndrome (DS) in Chile. Four tasks, based on established experimental paradigms modified to provide a game-like experience, were tested in 68 children with DS from 20 months to 12 years of age. We present evidence of reliability based on internal consistency and split-half analyses, with results ranging from adequate to excellent. Regarding validity, factorial and correlational analyses show evidence consistent with what was theoretically expected of internal structure, convergence, and divergence with other measures. Expected age trajectories were observed as well. Our data offer evidence that supports the use of tasks based on touch-screen devices for cognitive assessment in the population with DS. The tasks also have a low cultural load, so they could be validated and used in other contexts without the need for an adaptation process.

## 1. Introduction

Down syndrome (DS) is the most common genetic disorder in children and the most common genetic disorder associated with intellectual and developmental disability (IDD) [1,2,3]. Differences in prevalence have been reported between the United States, Europe, and Latin American countries like Chile, with rates of 1.4/1000 and 2.5/1000 live births, respectively [4]. The behavioral phenotype associated with this condition has been explored in several studies [5,6,7]. Some authors describe a general profile with specific characteristics [8,9,10], while others point to the existence of subgroups with different profiles of strengths and weaknesses [11,12,13].

Several studies suggest that cognitive abilities are dynamic and change during ontogenetic development in typical and atypical groups [6,14,15]. For this reason, it is possible to observe cognitive profile differences between age groups [14,16], as well as an important individual variability that has been indicated as a nuclear characteristic in the presence of a neurodevelopmental disorder (NDD) [17].

Previously published data points to an emergent phenotype with strengths and weaknesses in children with DS [7,18,19]. These studies suggest that the most common characteristic is relative strength in the visuospatial processing of information and weaknesses related to phonological working memory capacity [8,14]. Several authors point to the importance of memory systems in DS due to the close relation between this condition and Alzheimer’s Disease [20]; however, less information is available about this domain in children when compared to research in adults. Some authors have described specific difficulties in verbal short-term memory in people with DS, while non-verbal short-term memory seems to be a strong point in their cognitive profile [21].

Language has been identified as one of the most important weaknesses in DS, with a general delay and a dissociation between better comprehensive abilities versus more difficulties in expression abilities [22,23,24]. In contrast, the social dimension appears to be a strength, as some children with DS show adequate abilities to establish social relationships [25,26].

Regarding attentional processes, there are reports of different developmental trajectories for each subsystem in children with DS [27]. Several studies using tools to explore attention with tasks based on laboratory paradigms (in one case, even with a specific battery designed to explore the attentional subsystems [27]) have reported that selective attention follows a similar trajectory in children with DS and typical development (TD), in contrast with the trajectory of sustained attention which appears to have a slower rate and a lower ceiling in children with DS in comparison with TD peers [27,28,29,30]. The predictive relationship between attention and more complex cognitive abilities in DS has been well documented [30].

Visuospatial development in DS has been less studied. Previous research has compared visual and verbal abilities in children with DS and other genetic syndromes, and some studies have suggested that visuospatial abilities are a strength in DS [31]. Despite these results, this strength is relative as some studies conducted with toddlers with DS have shown problems with spatial cognition and space control using experimental paradigms such as the double-step saccade task [32].

Results concerning motor development are contradictory because, while it is common to think of this area as a strength compared to language development, some studies report difficulties in motor planning and praxis [33]. In school-aged children with DS, some authors have also observed difficulties in motor proficiency [34].

Finally, there is a significant amount of research regarding executive functions (EF) in preschool and school-age children with TD [35,36,37,38]. However, there is less information available about EF in DS, particularly from a developmental perspective. Previous results point to transversal weaknesses in this domain, indicating that this is an area with major constraints in cognitive development for people with DS [39,40,41,42], but the developmental trajectories for each component, as well as associations and dissociations in preschool-age, are not clear yet.

In the presence of a dynamic profile and with the specific characteristics of cognitive development described for DS, we need valid and reliable instruments for cognitive assessment in clinical settings. It is important to note that the use of traditional tests to assess cognitive abilities in people with NDD, and in particular with people with DS, has been criticized [13,43,44]. Floor effects are commonly observed in this population, and without observed variance in the assessments, it is difficult to identify individual profiles [45,46]. Cognitive assessment tests also tend to have high requirements in terms of oral communication, meta-cognition, monitoring, and other complex abilities, which make them unsuitable for assessing children with NDDs and show a need for new instruments that consider their characteristics.

Different groups have worked in the development of cognitive assessment tests for people with DS [1,47,48,49]. Some of these tools include technology as a way to avoid access barriers, others are available for research, and some have been used with children [50,51,52,53,54,55,56]. However, there are fewer available tests for the cognitive assessment of toddlers with DS, or those that can be used with children in a broader age range.

There is no doubt that this is an area with important advances in developed countries where two factors converge in favor of the DS community. First, the testing industry is well-developed in these countries, and professionals can assess different cognitive domains using several tests and batteries with robust evidence of reliability and validity according to international standards [57]. A different situation exists in developing countries where access to batteries with robust psychometric evidence is scarce. This situation also impacts the development of new instruments since there are not enough gold standard tests to establish, e.g., evidence of validity based on the relation with other variables. In developed countries, many aspects of professional practice are strictly regulated, making respect for copyrights and original material mandatory. In developing countries, such aspects are barely regulated, and test materials may be used inappropriately [58].

In the present study, we address the difficulties in cognitive assessment of children with DS using the design and testing of a battery of tasks based on games and technology. This battery has been developed to support clinical practice in Latin America, and the final package will be available for free for psychologists with specific training in cognitive assessment.

Based on traditional paradigms used mainly in laboratories for cognitive assessment of children with DS, we designed games that do not require complex oral language or meta-analytical abilities to reach the goals. Our objective was to develop tasks that provide strong evidence of reliability and validity for assessing attentional processes, visuospatial organization skills, and certain components of executive functions, namely working memory and inhibition. There is evidence indicating that these reported domains are reliable predictors of performance in cognitive development measures for children with intellectual and developmental disabilities (IDD) [5,8,29].

During the design and testing phases, we reviewed the literature on those domains to identify the best experimental paradigms used in our target group. To our knowledge, there are no specific tests to assess visuospatial abilities in toddlers and children with DS. Rather than specific instruments, common practice includes clinical protocols and natural observations for the assessment of gross motor abilities [59]. Regarding the assessment of attentional processes and behavioral inhibition in children with DS, traditional paradigms like the Continuous Performance test (CPT) and Sustained Attention to Response Task (SART) tasks are commonly used [60,61,62]. On the other side, experimental tasks such as the Dots task [63] and the Corsi Block-Tapping task [64] are frequently used for working memory assessments [21,65].

Our tasks were designed as tablet games since the literature suggests that games and technology are two elements that can help to create a “stealth assessment” situation [66] that is perceived by the child as a playful setting, thereby avoiding or decreasing frustration [67,68].

Our main goal was to demonstrate that this kind of cognitive assessment can reach acceptable levels of reliability and validity in toddlers and children with DS and could be used by clinicians in our cultural context. These tools can assist in conducting longitudinal or cross-sectional assessments across a broad range of ages. Moreover, they can demonstrate that the ability to obtain complex cognitive profiles is beneficial for the development of interventions and educational programs.

## 2. Materials and Methods

### 2.1. Participants

The sample included 68 children (38% female) with a medical diagnosis of Down Syndrome (all with non-disjunction variant), aged 20 to 146 months (M = 72.28, SD = 36.96). All children participated in early stimulation programs, and all were attending preschools or schools in the urban zones of Santiago, Chile. The sample was divided into four groups according to chronological age for the purposes of some of the statistical analysis (see Table 1). The average time the participants had spent in complementary therapies was 64.27 months (SD = 38.21 months; range: 6–145 months), and 40% of the participants 3 years of age or older were enrolled in special education schools. Table 1 presents frequencies for the demographic variables of the sample.

Children with neurological conditions other than DS, relevant medical conditions, previous identification of autism, or who were receiving complex medical treatment at the time of the assessment were excluded from the study. Other exclusion criteria included major surgery during the last three months before the assessment or a new pharmacological treatment in the initial phase.

As personal and family risk factors, we identified 52% of the children with heart diseases. The average duration of pregnancy for this group was 37.5 weeks, without differences between vaginal and cesarean section delivery. Only 6.6% of the parents received a prenatal diagnosis, and the mean maternal age of delivery was 37 years (SD = 6.60). All the developmental milestones of the children assessed were reported with delay by their caregivers.

Because our games were developed based on touch screen devices (i.e., tablets), we also conducted a survey about technology use by the children at home to account for previous experience using tablets or smartphones. All parents answered that their children have contact with touch screen devices at least five days each week. The main uses for the device were playing games (92%) and watching videos (53%). All parents reported maintaining a vigilant and interactive attitude with their child’s use of these devices.

### 2.2. Testing Procedure

This study was conducted in accordance with the ethical standards presented in the Helsinki Declaration. Ethical approval was given by the Ethical and Bio-ethical Committee at Pontificia Universidad Católica de Chile. Written consent was obtained from the parents of all participants. Informed assent was also used for children aged three or older, where the evaluator explained to the child the kind of activities they were going to be required to complete and asked for their assent to participate. If the child refused to perform the tasks, the assessment was suspended.

The first contact with the families and children with DS was established via organizations that work with the population. Interested families signed the informed consent and completed a socio-economic questionnaire. Participants then completed two assessment sessions of one hour each in a laboratory setting. One hundred and fifty-six families contacted our team, expressing their interest in our research. After the initial contact, 88 families attended the first assessment session, where their child was assessed with the Test de Aprendizaje y Desarrollo Infantil (Learning and Development Test; TADI) [69]. Ten families were not available for the follow-up session that included the tablet assessment, and they were excluded from the analyses.

Children aged three or younger were accompanied by one parent during the assessment; older children were accompanied by an adult if the child asked for their support. The assessments were conducted by psychologists who had previous work experience with children with IDD and neuropsychological evaluation. This team received specific training in behavioral management to deal with anxiety and behaviors that challenge.

The sessions were conducted using a fixed order: first, the traditional test (TADI), and then the tablet games, presented in the same order each time. At the end of each session, the evaluator completed an external criteria questionnaire (Questionnaire about attentional control and inhibitory behavior). The data obtained were digitalized in anonymous databases. All families received a written report three weeks after the sessions with information about how their child performed on the dimensions included in the neurodevelopmental scale used.

### 2.3. Instruments

Five instruments were used in this study: (a) a socio-demographic questionnaire; (b) TADI [69], a test designed to assess development in children from three months to five years of age; (c) BENDI, which is a set of four tablet games developed for the present study; (d) a questionnaire about attentional control and inhibitory behavior and; (e) an interview about the use of technology by the child. A detailed description of each instrument is presented.

#### 2.3.1. Socio-Demographic Questionnaire

Socio-demographic data were collected using the same form used in previous studies [70]. Questions include the age of the child, gender, educational level of the caregivers, type of school the child is attending, and data about clinical conditions and therapies.

#### 2.3.2. Test de Aprendizaje y Desarrollo Infantil (Learning and Development Test; TADI)

TADI [69] is a developmental battery based on the Bayley Scales of Infant and Toddler Development [71]. TADI was standardized for the Chilean population with a sample of 3200 children with TD and is suitable for children between three months to five years and eleven months of age. It assesses four dimensions: Cognition, Language, Motor, and Socio-emotional development. The authors of TADI present strong reliability and validity evidence for the test [69]. The TADI manual provides evidence of reliability via internal consistency coefficients explored via Cronbach’s Alpha, ranging between 0.8 and 0.9. Regarding validity evidence, the authors offer information based on test content using expert judgment, validity evidence based on the internal structure of the test via Confirmatory Factor Analysis with age-appropriate and scale-acceptable values, developmental progression analysis using the Sidak test with results within appropriate parameters, evidence of cultural content validity with results that rule out bias, and convergent validity evidence with other developmental scales. A previous study in Chile provided evidence for its use in children with DS [72].

#### 2.3.3. Battery for Neuropsychological Assessment in Intellectual Disability (Batería para la Evaluación Neuropsicológica en Discapacidad Intelectual-BENDI)

BENDI is a battery derived from the Test de Evaluación Neuropsicológica Infantil (TENI) [67]. The original battery was standardized for children with TD from 3:0 to 9:11 years of age. As a comprehensive battery, it provides a cognitive profile of general domains (attention, visuomotor development, language, memory, and executive functions). Despite good evidence of reliability and validity, TENI presented some problems for its use in children with intellectual disability, related to a complex set of verbal instructions in some of the tasks, the difficulty of its use in toddlers, visual information that distracted children, and a lack of appropriate performance feedback. For these reasons, our team worked to develop an adapted version suitable for toddlers and children with DS. This new instrument, called BENDI, is presented in this study.

BENDI, in its final form, includes four games: Big Fly, Firefly Hunt, Duno and the Worms, and Pop Pop Balloon. Each game collects information about several variables that are agglomerated into four general abilities: inhibitory control, attention, motor ability, and context memory. Along with these four games, other activities were designed during the research process: three oriented to assess working memory (one via phonological loop and two via visuospatial sketchpad), one designed as an adaptation of the Stroop test for children (Day and Night test) [73], and one oriented to explore cognitive flexibility. Those games showed floor effects in children under 3 years of age and were not included in the final battery.

Table 2 describes the games included in BENDI and their associated variables. All the activities include example items used by the evaluator to model the task and practice items oriented to verify the level of comprehension of the instructions. However, if the child has a good level of oral comprehension, standardized instructions are also available in the Manual. The evaluator can decide the order of application and whether oral instructions are needed.

#### 2.3.4. Questionnaire about Attentional Control and Inhibitory Behavior

Attentional control and inhibitory behavior during the assessment were assessed with a checklist adapted from the Leiter International Performance Scale–Revised [75]. This Questionnaire has been previously used in two Chilean samples of preschoolers with TD [76,77] and explores two dimensions, attentional control and inhibitory behavior, each with five questions. The abilities explored include focused and sustained attention, impulse control, cooperation, interest in the activities, emotion control, and motor activity. Three indexes are calculated from the scale: inhibitory control (min = 5, max = 20), attentional control (min = 5, max = 20), and total score (min = 10, max = 40). The internal consistency of the scale in this study, analyzed using Cronbach’s Alpha, was 0.902.

#### 2.3.5. Technology Use Survey

A survey was applied to all the parents in this study. This survey included questions about technology use, frequency, and preferences in their children.

### 2.4. Statistical Analysis

We obtained descriptive information about the participants’ socio-demographic and clinical characteristics. Then, a descriptive analysis was performed for each variable, including mean, standard deviation, skewness, and kurtosis. Next, results about general performance and specific information about floor effects were calculated for each task. Evidence of reliability was calculated using Cronbach’s Alpha for Pop Pop Balloon, Duno, the Worms, and Firefly Hunt, while internal consistency for Big Fly was analyzed using the split-half method due to the characteristics of the game. Evidence of validity was assessed using correlational analysis between the variables from BENDI, the analysis of the mean performance change by age, and correlations between BENDI and other measures (TADI and the Questionnaire about attentional control and inhibitory behavior) to assess the relationship with other variables (convergent and discriminant validity). Finally, a variance analysis was used to evaluate predictive validity. Analyses were performed in SPSS 24 [78] and R 4.2.1 [79] statistical packages.

## 3. Results

Our results can be synthesized in two main aspects: (1) cognitive assessment with a traditional battery reflects a delay in toddlers and children with DS without specific evidence to support a disharmonic profile, and (2) our results in the process of developing tablet games for cognitive assessment supported previous data about the benefits of using technology as a way to deal with floor effects [67].

Results obtained with TADI [69] indicate a general delay in development. These analyses considered the age subgroups. Raw scores were below the expected age scores, and T scores were in the “extreme risk” range, that is, the test performance floor. The averages by age group for TADI appear in Table 3.

We analyzed the distribution and floor effect for each variable included in the final package of tablet games (see Table 4) for the entire sample (*n* = 68). The floor effect was analyzed based on the percentage of children that obtained the lowest possible value for each of the calculated variables. Five tasks originally included in BENDI and designed to assess working memory, cognitive inhibition, and cognitive flexibility were discarded because more than 50% of the sample showed floor effect. In some of these tasks, children had problems understanding the instructions as well. In the four final tasks, 8.8% of children were unable to complete Big Fly, which is the easiest game, and 38.2% were unable to complete Duno and the Worms, the most difficult one.

We also analyzed skewness and kurtosis levels, and only two variables were extreme: the number of times that the child touched the screen (Touches) and how many times the child touched the screen when there was not a worm (Impulsivity) in Duno and the Worms. In the first case, this was caused by a group of children touching the tablet repeatedly after answering the item. In the second case, most children did not behave impulsively, and the indicator reflected this by assigning a score of 0. We chose to keep both variables for further analysis because the results were consistent with the observed behavior.

### 3.1. Evidence of Reliability

Evidence of reliability was estimated for each game according to international standards [57]. We estimated internal consistency for three of the tasks. For Duno and the Worms, the estimate was 0.638 (Adequate); in Firefly, it was 0.914 (Excellent); and in Pop Pop Balloon, it was 0.959 (Excellent). For Big Fly, the correlation index in the split-half analysis was 0.716 (Good).

### 3.2. Evidence of Validity

We offer evidence of validity in three dimensions: content analysis, internal structure, and relationship with other variables (convergent and divergent), as required by current standards [57]. The first evidence source comes from content analysis and design during TENI implementation and validation. Both tests, TENI and BENDI, were designed following cognitive neuropsychology principles. Each game is focused on assessing specific cognitive domains and measuring them as specifically as possible, according to the modularization and specialization expected at each age.

The proposed activities incorporate characteristics of classic experimental paradigms that have been previously tested in persons with disabilities. Innovation, in this case, comes both from the use of touch devices (and their usability gains) and the implementation of instructions with modeling and practice, as well as verbal instructions for the tasks designed to consider the needs of children with DS.

Evidence related to the tasks’ internal structure is offered via correlational analysis, trajectories of performance by age, and exploratory factor analysis. The correlation matrix shows strong and significant correlations between several sets of variables (Table 5). These are used to glean three aggregates, which point to three components: motor coordination, attentional control, and inhibitory control. Strong significant correlations ranging from 0.59 (*p* < 0.001) to 0.815 (*p* < 0.001) were found between the tasks in each aggregate, while no significant correlations with variables from the other aggregate were found between the variables of the inhibitory control aggregates and the variables from the other two aggregates. The set of variables measuring inhibitory control (touches, commissions, and impulsivity in Duno) are correlated. Finally, the variables that can be associated with attentional control are also strongly and significantly correlated with each other.

Trajectories by age for each variable correspond with the expected theoretical trajectories, i.e., improvements in both performance and accuracy for each task. All variables show progressive and significant changes and no ceiling effects.

We checked for the underlying factorial structure. The Kaiser-Meyer-Olkin measure of sampling adequacy was 0.648, and Bartlett’s test of sphericity was significant (χ^2^ (66) = 407.40, *p* < 0.001). We performed an exploratory factor analysis using Maximum Likelihood as the extraction method and a Varimax rotation with Kaiser normalization. The extraction yielded a three-factor solution (Table 6) that explains 43.75%, 17.52%, and 14.77% of the variance, respectively. The variable groupings also suggest factors for motor coordination, attentional control, and inhibitory control.

As further evidence of validity, we also looked at the relationship with other variables: TADI and the Questionnaire about attentional control and inhibitory behavior. To explore the convergent and divergent validity as well as the tasks’ predictive capability, we analyzed the correlation matrix between BENDI and TADI (Table 7). All game variables and TADI scores have strong and significant correlations ranging from 0.771 (*p* < 0.001) to 0.519 (*p* < 0.001). All correlations have the expected direction: accuracy and performance variables have positive values, while impulsivity and inattention have negative values. Finally, we analyzed the correlations between each variable and the Questionnaire about attentional control and inhibitory behavior (Table 8). We used the scores obtained in each scale of the Questionnaire about attentional control and inhibitory behavior (inhibitory control and attentional control) and in the total scale to group children in quartiles for each score (from Lowest = 1 to Highest = 4) and used the group as a factor in a one-way analysis of variance for each game variable.

## 4. Discussion

With this study, we aimed to offer a set of tasks in the form of tablet games oriented to help with the cognitive assessment of children with DS in Chile. Four games that assess attentional control, visuomotor development, inhibitory control, and contextual memory in toddlers and children with DS are presented. These abilities were selected instead of those commonly associated with the cognitive phenotype based on the identified need for instruments that allow the assessment of basic and early cognitive functions.

In these specific tasks, we incorporated the use of a tablet device as the platform to carry out the assessment, trying to exploit the apparent advantages of touch devices to improve the assessment setting, supported using proven experimental paradigms. These advantages include interactive environments facilitated by multi-sensorial activities with more probabilities to enhance attentional control than traditional tasks. According to Cristia et al. [80], children with TD exhibit high levels of usability in touch screens. Our data suggest that the appropriate use of touch devices among toddlers and children with DS is also high and that they have early experiences with these kinds of devices.

Reliability and validity evidence for the tasks has been reviewed, with results indicating that the four BENDI tasks’ levels range from adequate to excellent in reliability measurements. Regarding evidence of validity, we provide evidence of the internal structure validity of the instrument based on the correlations between the BENDI variables and each of its tasks, exploratory factor analysis, and the relationship between task performance and age. The correlation matrix between the BENDI variables is consistent with what is theoretically expected, also considering what would be expected according to the three factors identified in the factor analysis. In this line, the exploratory factor analysis suggests a three-factor structure that would correspond to motor coordination, attentional control, and inhibitory control. BENDI was developed in search of an instrument that allows the evaluation of basic cognitive skills, which may be relevant domains for the development of more complex cognitive skills. The internal structure of BEDI is consistent with what other authors have suggested regarding these domains as relevant to cognitive development in people with DS [81,82,83].

The evidence of convergent and divergent validity was obtained from an analysis of the relationship between the development scale (TADI), the external criterion questionnaire for the assessment of attention and inhibition behavior, and BENDI. The BENDI variables’ correlation matrix with the evaluation of motor, cognitive, language, and socio-emotional development using TADI shows that there is a close relationship between the development of children with DS and the variables explored in BENDI, except for those that evaluate attentional control and behavioral inhibition in the Duno and the Worms tasks, although these correlate with the external criterion questionnaire for behavioral inhibition and attentional control. We understand behavioral inhibition as the ability to delay a behavior, allowing control over the behavioral action to reach a pre-established goal [36]. The behaviors checklist used includes items related to behavioral inhibition between tasks, the level of cooperation with the evaluator represented by behaviors like eye contact during the instructions and gaze towards the task, the disposition to participate in the proposed activity, following instructions, and emotional modulation and regulation. These behaviors have significant and high correlations with better results in the developmental test (TADI) and better performances in the activities.

There are two plausible hypotheses to explain the relation described between behavioral inhibition and performance in cognitive tasks. The first hypothesis is that children with better abilities to inhibit behavior are better at inhibiting preponderant responses, halting ongoing responses, and managing environmental interference, which facilitates the development of working memory, self-regulation of effect, and speech internalization. This, in turn, impacts performance in high-level functions, represented in Executive Functions [84]. The second hypothesis seems less complex but is harder to demonstrate: perhaps children with higher cognitive abilities are better at regulating their behavior. More research is needed to answer this question.

The Questionnaire about attentional control and inhibitory behavior was also used to evaluate attentional control. The behaviors observed included the time the child stays on the task, attention during instructions and demonstrations, observed effort to give a correct answer, sustained goal-directed actions even during repetitive tasks, and care for devices and supporting materials. These behaviors also had significant correlations with the scores in TADI. These findings indicate that there is a cognitive action of voluntary attentional control that develops with age, which is another important predictor of cognitive development. These results suggest that as behavioral inhibition and attentional control are closely related to childhood development, an early focus in the context of therapeutic interventions for toddlers and children with DS, these abilities could be as important as the longstanding focus on motor and language interventions. Future studies should address this issue.

As was stated in the introduction, there is low access to valid and reliable instruments to assess cognitive variables in Latin America. In a few countries in the region (e.g., Brazil, Mexico, Argentina, and Chile), there is an industry dedicated to the development and adaptation of psychological assessment tools, and it is common practice to use foreign tests that lack cultural adaptations, even when the evaluated variables may be impacted by culture or language. This makes it imperative to have instruments with a low cultural load and evidence of adequate psychometric qualities for neuropsychological clinical practice in the region, especially when working with populations with atypical development, for whom traditional instruments are often not suitable. We hope that the development of BENDI contributes to meeting this need. With the present research, we cannot affirm that BENDI can be used outside the context in which it was developed, but its characteristics allow its psychometric qualities to be studied in other countries with little or no modification.

The availability of tests like BENDI, which incorporate the characteristics of children that can invalidate traditional assessment tests or make them impossible to use, has a practical impact. Such tests allow for the acquisition of a cognitive profile for children who are typically deemed “non-assessable” or exhibit floor effects. Creating a cognitive profile for the child can assist clinicians in gathering information about their abilities, facilitating intervention design, and enabling measurement of these interventions’ impact.

The present study has several limitations. First, the sample size is appropriate to present psychometric data about the tasks and is large compared to some previous studies, but it is insufficient to offer population norms. The tasks in their current form could be useful in clinical settings as an initial approach to assess performance in the abilities included, but they are not appropriate for identifying profiles or for intergroup comparisons.

Second, parents reported previous contact with touch devices for all the participants. This previous experience was important for our study because we wanted to be sure that the platform would not impose a barrier for the assessment, but it could be a problem if previous contact with touch screens produces a positive bias towards performance. However, previous research with preschoolers comparing performance in a task based on touch screens versus performance in traditional pencil-paper tasks showed no biases [85]. As previously mentioned, the use of these devices is also highly intuitive and does not require previous complex training.

Finally, we could not gather information about incremental validity and relationships with other variables specific to our tasks. As reported, there is a difficulty with the use of traditional tests with children with IDD, characterized by the floor effect. This situation created difficulties for comparisons with some gold standards.

## Figures and Tables

**Table 1:** Demographic characteristics of participants’ age groups.

Age Group	*n*	Age	Girls: Boys	Stimulation Duration (Months)
		Mean (SD)		Mean (SD)
≤5 years	32	2.94 (1.46)	13: 19	36.33 (18.41)
6–7 years	18	6.56 (0.51)	7: 11	73.69 (23.99)
8–9 years	10	8.50 (0.53)	6: 4	95.00 (28.08)
≥10 years	8	10.88 (0.84)	5: 3	123.00 (33.75)

**Table 2:** Tasks and their dependent variable.

Game	Description	Variables
Big Fly	This activity assesses visuospatial coordination via eye-hand coordination. Flies move across the screen, and the child must squash them with a finger. When the child touches the fly, there is a sound, and the fly is squashed. If the child touches the tablet but not the fly, the fly stops for a moment, and another sound is played. The game updates the difficulty level dynamically according to the child’s performance. It has two example items, two practice items, and a three-minute evaluation phase.	- Hits: Total flies squashed. - Perfect Hits: Total flies squashed without previous tries. - Hits per Fly: Ratio between hits and flies shown. - Hits per Touch: Ratio between hits and total touches.
Duno and the Worms	This test is based in the continuous performance test [60]. There is a monster (Duno) at the bottom of the screen, and apples fall from the top. Every time an apple falls, the child must touch the screen if a worm appears inside it. Every action has feedback; failure and success are represented in Duno’s face. The game includes 3 min for instructions and practices, and 3 min for the evaluation phase.	- Touches: Total touches. - Correct Hits: Total of touches made when a worm was present. - Commissions: Total of touches made when a worm was not present. - Impulsivity: Ratio of incorrect responses to nontargets to the number of nontargets - Presented, minus the number of anticipatory responses made to nontarget stimuli. - Inattention: Ratio of correct responses to targets, divided by the number of targets presented, minus the number of anticipatory responses to targets.
Pop Pop Balloon	A traditional shifting attentional paradigm [74] was modified for this task. A balloon appears from the top, either on the left or the right of the screen, and the child has to pop it by touching it. Each change in the side on which the balloon appears forces an attentional shift. The game has two screens for instructions and practice and a 3 min evaluation phase.	- Hit Ratio: Ratio between total correct pops and total balloons shown. - Shift Correct Ratio: Ratio between total correct pops after a shift and total shifts.
Firefly Hunt	A visual memory task where a nocturnal landscape is presented to the child; a firefly then appears, stays on screen for a short time, and then disappears. The child must touch the place where the firefly just appeared. The game has 3 example items, 6 practice items, and 20 evaluation items.	- Hit Ratio: Ratio between hits and fireflies shown.

**Table 3:** TADI scores by age group.

Age Group	Dimension	Raw Score Mean (SD)	Raw Score Range	Standard Score Mean (SD)	Standard Score Range
≤5 years	Cognition	19.8 (6.2)	10–30	31.0 (7.1)	23–48
	Language	21.4 (5.1)	12–35	33.3 (10.8)	23–57
	Motor	20.4 (6.1)	9–33	26.7 (5.7)	23–42
	Socioemotional	27.4 (5.2)	19–40	33.8 (12.5)	23–57
6–7 years	Cognition	28.9 (5.6)	21–41	29.6 (1.6)	29–34
	Language	30.1 (6.2)	19–44	23.8 (3.3)	23–37
	Motor	31.8 (5.1)	18–43	24.6 (5.2)	23–45
	Socioemotional	33.2 (4.3)	25–40	23.2 (0.7)	23–26
8–9 years	Cognition	35.1 (3.1)	30–40	29.3 (0.9)	29–32
	Language	36.3 (4.5)	30–45	24.6 (5.1)	23–39
	Motor	35.0 (5.2)	26–41	28.2 (6.6)	23–39
	Socioemotional	35.7 (4.1)	29–42	24.2 (2.1)	23–29
≥10 years	Cognition	38.9 (7.0)	27–45	35.0 (5.3)	29–42
	Language	38.9 (8.8)	28–51	32.6 (10.7)	23–53
	Motor	39.6 (3.7)	35–44	35.4 (10.6)	23–49
	Socioemotional	37.5 (7.5)	26–47	27.0 (5.9)	23–37

**Table 4:** Distribution data for each measure.

Measure	*n*	% Not Completed	% Floor	Mean	SD	Range	Skewness	Kurtosis
TADI	68	0						
Cognition			0	26.68	9.19	0–45	0.13	−0.79
Language			0	27.93	8.87	0–51	0.46	−0.38
Motor			0	27.84	9.14	0–44	−0.17	−0.89
Socioemotional			0	31.35	6.4	0–47	0.2	−0.48
Big Fly	62	8.80						
Hits			0	35.95	14.58	0–58 ^a^	−0.46	−0.86
Perfect Hits			6.5	25.82	16.36	0–58 ^a^	−0.16	−1.11
Hits per Fly			0	0.79	0.19	0–1	−0.85	−0.35
Hits per Touch			0	0.54	0.28	0–1	−0.23	−1.13
Duno	42	38.20						
Touches			11.9	10.29	15.58	0–92 ^a^	3.99	18.84
Correct Hits			21.4	2.88	2.12	0–6	−0.01	−1.34
Commissions			4.8	2.86	3.31	0–12	1.35	1.25
Impulsivity			n/a	1.12	2.01	0–9 ^a^	2.85	8.4
Inattention			n/a	0.21	0.17	0–0.5 ^a^	0.38	−1.09
Firefly Hunt	51	25.00						
Hit Ratio			0	0.6	0.29	0–1	−0.44	−0.8
Pop Pop Balloon	57	16.20						
Hit Ratio			0	0.67	0.31	0–1	−0.63	−1.08
Shift Correct Ratio			3.5	0.7	0.31	0–1	−0.82	−0.62

^a^ This variable has no fixed upper bound. The reported value is the maximum value reached by a subject.

**Table 5:** Correlations between variables.

	Big Fly				Pop Pop Balloon		Firefly Hunt	Duno				
	Hits	Perfect Hits	Hits per Fly	Hits per Touch	Hit Ratio	Shifts Correct Ratio	Hit Ratio	Touches	Commissions	Impulsivity	Correct Hits	Inattention
Big Fly												
Hits	1	0.939 **	0.942 **	0.767 **	0.815 **	0.790 **	0.696 **	−0.172	−0.057	−0.234	0.659 **	−0.441 **
Perfect Hits	0.939 **	1	0.882 **	0.887 **	0.794 **	0.769 **	0.763 **	−0.152	−0.01	−0.223	0.659 **	−0.445 **
Hits per Fly	0.942 **	0.882 **	1	0.762 **	0.838 **	0.811 **	0.722 **	−0.228	−0.041	−0.274	0.667 **	−0.417 **
Hits per Touch	0.767 **	0.887 **	0.762 **	1	0.736 **	0.730 **	0.691 **	−0.178	−0.041	−0.208	0.513 **	−0.297
Pop Pop Balloon												
Hit Ratio	0.815 **	0.794 **	0.838 **	0.736 **	1	0.974 **	0.677 **	−0.033	0.096	−0.064	0.591 **	−0.458 **
Shifts Correct Ratio	0.790 **	0.769 **	0.811 **	0.730 **	0.974 **	1	0.661 **	−0.062	0.106	−0.058	0.529 **	−0.389 *
Firefly Hunt												
Hit Ratio	0.696 **	0.763 **	0.722 **	0.691 **	0.677 **	0.661 **	1	−0.137	0.011	−0.171	0.594 **	−0.362 *
Duno												
Touches	−0.172	−0.152	−0.228	−0.178	−0.033	−0.062	−0.137	1	0.742 **	0.877 **	−0.184	−0.439 **
Commissions	−0.057	−0.01	−0.041	−0.041	0.096	0.106	0.011	0.742 **	1	0.870 **	−0.128	−0.344 *
Impulsivity	−0.234	−0.223	−0.274	−0.208	−0.064	−0.058	−0.171	0.877 **	0.870 **	1	−0.25	−0.227
Correct Hits	0.659 **	0.659 **	0.667 **	0.513 **	0.591 **	0.529 **	0.594 **	−0.184	−0.128	−0.25	1	−0.757 **
Inattention	−0.441 **	−0.445 **	−0.417 **	−0.297	−0.458 **	−0.389 *	−0.362 *	−0.439 **	−0.344 *	−0.227	−0.757 **	1

** *p* < 0.001; * *p* < 0.01.

**Table 6:** Rotated factor matrix for game measures’ EFA.

	Factor ^a^		
Measure	1	2	3
Big Fly Hits	0.957		
Big Fly Perfect Hits	0.944		
Big Fly Hits per Fly	0.936		
Big Fly Hits per Touch	0.836		
Pop Pop Balloon Hit Ratio	0.834		
Pop Pop Balloon Shifts Correct Ratio	0.820		
Firefly Hunt Hit Ratio	0.572		
Duno Touches		0.932	
Duno Commissions		0.753	
Duno Impulsivity		0.633	
Duno Correct Hits	0.334		0.911
Duno Inattention		−0.425	−0.865

^a^ Loadings with absolute values below 0.3 were removed for readability.

**Table 7:** Correlations between TADI and game measures.

	TADI			
	Cognition	Language	Motor	Socioemotional
Big Fly				
Hits	0.733 **	0.683 **	0.729 **	0.709 **
Perfect Hits	0.771 **	0.729 **	0.737 **	0.731 **
Hits per Fly	0.697 **	0.648 **	0.743 **	0.669 **
Hits per Touch	0.651 **	0.634 **	0.639 **	0.654 **
Pop Pop Balloon				
Hit Ratio	0.637 **	0.644 **	0.685 **	0.636 **
Shifts Correct Ratio	0.652 **	0.647 **	0.691 **	0.647 **
Firefly Hunt				
Hit Ratio	0.716 **	0.626 **	0.745 **	0.519 **
Duno				
Touches	−0.254	−0.215	−0.257	−0.185
Commissions	−0.026	−0.061	0.036	0.022
Impulsivity	−0.201	−0.198	−0.072	−0.119
Correct Hits	0.475 **	0.478 **	0.502 **	0.469 **
Inattention	−0.200	−0.226	−0.269	−0.257

** *p* < 0.001.

**Table 8:** Between groups, tests of game measure scores by lowest and highest performance quartiles in the Questionnaire about attentional control and inhibitory behavior.

	F (1)	*p*	η^2^
**Inhibitory Control**			
Big Fly			
Hits	3.130	0.095	0.155
Perfect Hits	6.107	<0.05	0.264
Hits per Fly	2.005	0.175	0.105
Hits per Touch	6.676	<0.05	0.282
Pop Pop Balloon			
Hit Ratio	5.568	<0.05	0.271
Shifts Correct Ratio	3.863	0.068	0.205
Firefly Hunt			
Hit Ratio	0.656	0.432	0.045
Duno and the Worms			
Touches	10.429	<0.05	0.487
Commissions	3.612	0.084	0.247
Impulsivity	0.747	0.408	0.070
Correct Hits	9.995	<0.05	0.476
Inattention	9.384	<0.05	0.460
**Attentional Control**			
Big Fly			
Hits	13.031	<0.001	0.352
Perfect Hits	12.100	<0.05	0.335
Hits per Fly	14.850	<0.001	0.382
Hits per Touch	10.755	<0.05	0.309
Pop Pop Balloon			
Hit Ratio	22.231	<0.001	0.514
Shifts Correct Ratio	17.360	<0.001	0.453
Firefly Hunt			
Hit Ratio	0.539	0.473	0.033
Duno and the Worm			
Touches	2.405	0.140	0.131
Commissions	0.463	0.506	0.028
Impulsivity	0.075	0.789	0.005
Correct Hits	21.230	<0.001	0.570
Inattention	2.281	0.150	0.125
**Total**			
Big Fly			
Hits	7.369	<0.05	0.290
Perfect Hits	9.749	<0.05	0.351
Hits per Fly	5.422	<0.05	0.231
Hits per Touch	7.537	<0.05	0.295
Pop Pop Balloon			
Hit Ratio	9.341	<0.05	0.384
Shifts Correct Ratio	8.122	<0.05	0.351
Firefly Hunt			
Hit Ratio	0.423	0.528	0.034
Duno and the Worm			
Touches	1.702	0.217	0.124
Commissions	0.004	0.950	0.000
Impulsivity	0.021	0.889	0.002
Correct Hits	30.656	<0.001	0.719
Inattention	1.904	0.193	0.137

## Data Availability

The data that support the findings of this study are available on request from the corresponding author. The data are not publicly available due to containing information that could compromise the privacy of research participants.
